# Injurious information propagation and its global stability considering activity and normalized recovering rate

**DOI:** 10.1371/journal.pone.0258859

**Published:** 2021-10-28

**Authors:** Chaoqian Wang, Ziwei Wang, Qiuhui Pan

**Affiliations:** 1 School of Physics, Dalian University of Technology, Dalian, China; 2 School of Innovation and Entrepreneurship, Dalian University of Technology, Dalian, China; 3 Department of Computational and Data Sciences, George Mason University, Fairfax, VA, United States of America; 4 School of Mathematical Science, Dalian University of Technology, Dalian, China; Beijing University of Posts and Telecommunications, CHINA

## Abstract

This paper establishes a compartment model describing the propagation of injurious information among a well-mixed population. We define the information’s injuriousness as the people practicing the information being injured and leaving the system. Some informed people practice the information and are active, while others do not practice and are inactive. With the recovery resources fixed, the two groups of informed people’s recovering rates are normalized considering the information features. The stability of the nonlinear system is thoroughly studied. Analyzing the reproduction number of the injurious information, we find that in general parameter space, when there are people in an informed compartment, it is not always necessary to consider their recovery resource allocation. Instead, only when their proportion reaches a critical point should it be allocated. Unless the people in an informed compartment form a certain proportion, we can take a laissez-faire attitude towards them. In a more realistic parameter space, once inactive informed people exist, they should be allocated recovery resources. On the one hand, when the recovering rate rises, the focus on both groups of informed people is necessary for more situations. On the other hand, when the rate of active informed people leaving the system rises, ignoring active informed people benefits removing the injurious information in more cases. The model provides qualitative ways in the scenarios of removing injurious information.

## 1. Introduction

Kermack and McKendrick [1] first proposed an SIR compartment model depicting epidemic propagation. In the classical SIR model, according to the heterogeneity of people’s state of the epidemic, the well-mixed population is divided into three compartments: susceptible (*S*), infected (*I*), and recovered (*R*). Assuming the infection rate is *α*, the recovering rate is *ω*. Then, in a unit time, there are *αSI* susceptible becoming infected, and *ωI* infected becoming recovered. The SIR model being not suitable for all kinds of epidemics, a variety of classical epidemic models (e.g., the SEIR model) were proposed concerning the characteristics of different epidemics. In the classical SEIR model, compartment *E* is exposed, and after infection, the susceptible will not immediately have infectivity but will be exposed first. Although the mathematical form of compartment models seems simple, the research on their deep mathematical essence has never been interrupted. For instance, in 2020, the accurate solutions of the SIR epidemic model [2–4] and the SEIR epidemic model are found [5]. In general, the compartment models are usually idealized. While some works are data-driven [6], most of others can guide some epidemic prevention and control problems in the real world only at the qualitative level but without real data [7–10].

The stability analysis attaches tremendous significance to compartment models. With the development of modern control theory, it is incorporated into the research framework of nonlinear dynamics. For the local stability, van den Driessche and Watmough [11] proposed a general method for finding the basic reproduction number, which can be used to judge the local stability of the epidemic-free equilibrium. Lyapunov’s Second Method and Lasalle’s Invariance Principle [12, 13] are essential mathematical tools for global stability analysis. The global stability of the classical SEIR model was analyzed by Li et al. [14, 15]. Various additional mechanisms were then implemented to the SEIR model with the global stability completely analyzed; for example, the immigration of different compartments [16], the nonlinear transmission rate [17, 18], the distributed delays [18], the discontinuous treatment [19], the age-dependent latency and relapse [20], the non-monotone incidence rates [21–23]. Global stability analysis was also carried out in epidemic models with real backgrounds [24, 25]. Some literature has summarized the general global stability analysis methods for several classical compartment models [26, 27].

There has been considerable interdisciplinary research on the epidemic compartment model, such as the rumor spreading and radicalization. In 1964, Daley and Kendall [28] compared the similarities between epidemics and rumors and proposed to use the epidemic compartment model to describe the spread of rumors. Based on the classical compartment of rumor propagation, a series of mechanisms were investigated. For example, Zhao et al. [29, 30] implemented the mechanism of forgetting rate to rumor spreading model, Wan et al. [31] studied the rumor propagation model with demographics on scale-free networks, Hu et al. [32, 33] built rumor spreading models considering different reactions of different people towards rumors, and Xiao et al. [34] combined the rumor spreading model with evolutionary games. In the study of rumor propagation, the stability analysis of the compartment models was also detailly carried out [31–33]. Like modeling the rumor spreading, Galam and Javarone [35] modeled the phenomena of radicalization in heterogeneous populations. Similarly, Chuang et al. [36, 37] investigated a bistable belief system for radicalization within sectarian conflict [36] and the impact of age-structured social interactions on radicalization [37]. Santoprete et al. [38–40] created a series of compartment models to study the strategy of de-radicalization. In the study of the process of radicalization, stability analysis was carried out [35, 39, 40]. In the framework of a nonlinear continuous system [41] or discrete system [42], the compartment model can describe the propagation of general information, ideology, or opinion as long as it has a similar law transmission.

This paper proposes a compartment model depicting information propagation based on the classical SEIR epidemic compartment model. A similarity and a difference between the propagation of information and epidemics are respectively considered. First, we label the information discussed in the paper as “injurious information.” Similar to epidemics, its injuriousness lies in the dynamical characteristics that the people who are informed flow out of the system. For example, in influenza pandemics, the information of advocating the harmfulness of vaccines causes the informed people who practice the information (choose not to vaccinate) to be injured in health, thus flowing out of the system (e.g., hospitalization or death). On the contrary, the other part of the population may not practice this type of information (still choose to vaccinate), even if they are informed. Most medically relevant opinions or rumors can be considered in this category. For instance, opinions such as violent extremism and terrorism can also be considered in this category, whose practitioners may leave the system because of their own deaths or because of social isolation (treatment by law enforcement). As described above, this paper considers the different practical attitudes of people to injurious information. It divides them into active informed (those who practice the information) and inactive informed (those who do not practice it), and this distinction between the two depends on the population properties, including but not limited to the age structure and education level.

Different from the epidemic models, secondly, we consider the normalization of these two groups of people’s recovering rate. For the recovery process of epidemics, the use of medical resources (e.g., ventilators) is restricted to the number of people; therefore, the number of people using medical resources per unit time (i.e., the recovering rate multiplied by the number of infected people) is constant. The normalization of the recovering rate among heterogeneous infected people is meaningless. But, concerning information spreading, the recovering rate of heterogeneous informed people turning into recovered people can be normalized. This is because that, the popularization of knowledge to the public by the government public welfare organization or folk media is based on platforms such as the Internet or other traditional media; therefore, there is no extra cost for promoting recovery resources (e.g., rumor refutation notice) to more people. Unlike in the epidemic treatment, the medical resources for a certain number of infected people correspond to a certain amount of cost, in the injurious information removing, it is the recovering rate for a particular group of people on the information platform that corresponds to a certain amount of cost. Then, in the exposure of recovery resources to the crowd, we need to consider the push to different groups of people, not to a fixed number of people. In other words, assuming fixed recovery resources, the recovering rate between different groups is normalized.

In addition to the basic assumptions in dynamics, it should be noted that the “injurious information” in this paper does not involve value judgment, but only refers to the information that leads the informed people objectively flowing out of the system for any reason. Once a kind of information has such dynamical phenomena in its informed people, this paper’s model could be applied.

This paper will first complete the local and global stability analysis of the compartment model and then study the qualitative strategies on removing the injurious information from the perspective of the basic reproduction number of the information.

## 2. Equations

Consider a well-mixed population, where a piece of injurious information spreads (we will refer to this specific injurious information as “the injurious information”). Based on the heterogeneity of people’s state of the injurious information, the population is divided into five compartments.

- Susceptible (*S*), those who have not yet received and heard the injurious information.
- Exposed (*E*), those who have received the injurious information, but are pondering over it and will not spread it further for the time being (e.g., are in a state of “neutral attitude” [32, 33]).
  The informed compartments consist of those who have grasped the information and can spread it. This paper has two informed compartments. The difference between the two may be due to the age structure and education level.
- Active informed (*I*_+_), those who are active towards the injurious information, thus flowing out of the system at a specific rate. For example, those who practice the rumors about medicine and health after listening to it and are hurt in health, or those who practice the extremist information and are dealt with by the security services.
- Inactive informed (*I*_−_), those who are inactive towards the injurious information, such as those who believe in the information but are inactive in behavior, thus not hurt by it.
- Recovered (*R*), those who have recovered from the injurious information. We set this compartment because people’s attention cannot stay on the same information forever; examples include forgetting [29, 30], resisting [32, 33], being treated [40], or only no longer spreading it [28].

**Fig 1:**
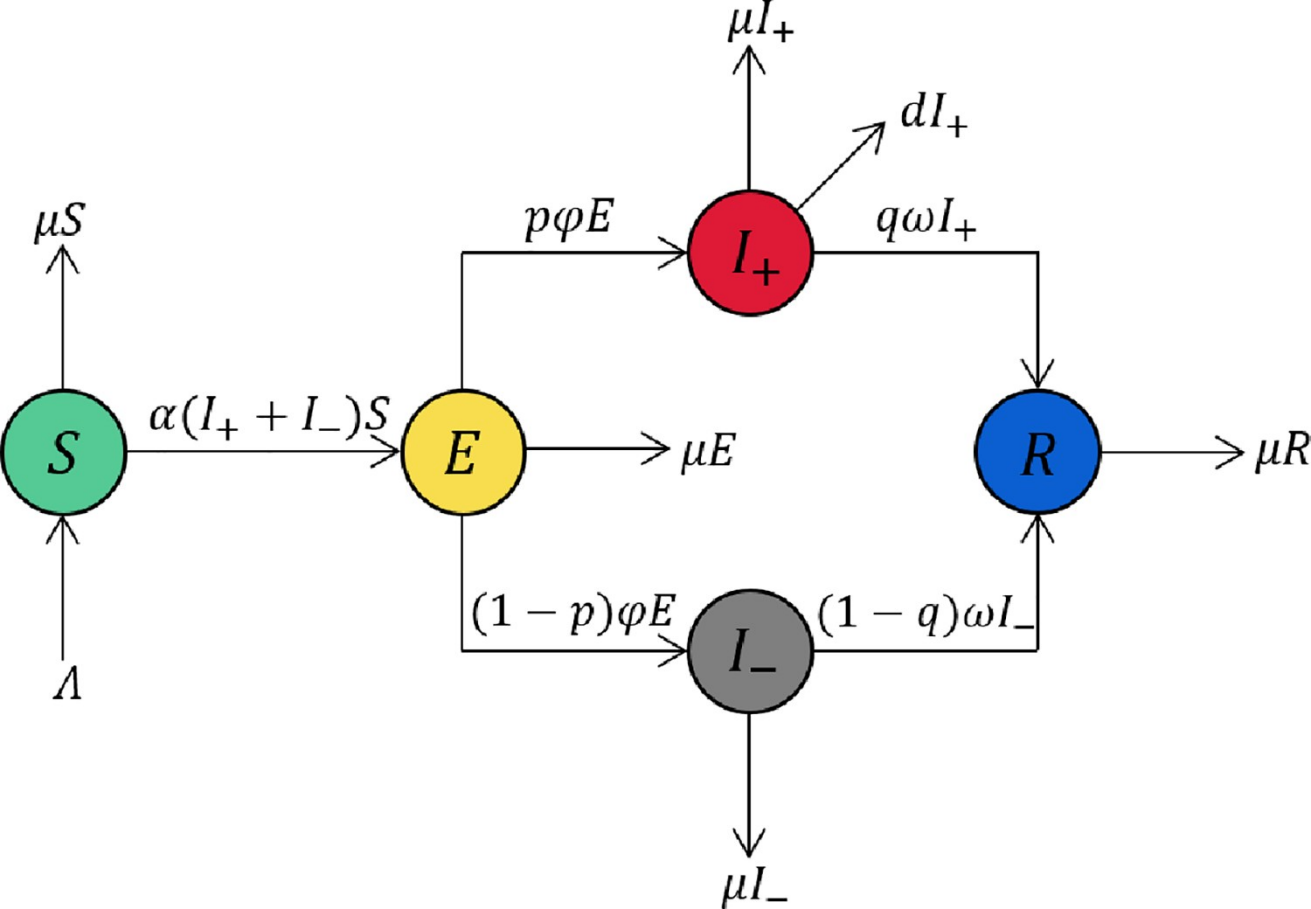
The flow of the population.

The dynamics of the population are demonstrated in Fig 1. The symbols *S*, *E*, *I*_+_, *I*_−_, and *R* are the population in five compartments. The algebraic expression on the arrow represents the number of people who flow from one compartment to another in unit time.

The injurious information is transmitted through human-to-human contact. It is the informed people who spread the information in the system. We assume the active and inactive informed people spread the information at the same rate, denoted by *α*. Within a unit time, *S* susceptible people contact the informed people numbered (*I*_+_ + *I*_−_) and are informed at a rate of *α*. Therefore, *α*(*I*_+_ + *I*_−_)*S* people flow from compartment *S* to compartment *E* in unit time.

After receiving the information, the susceptible people need time to grasp the information before spreading it (we call such a state an exposed state). We denote the rate at which exposed people access the dissemination capability by *φ*; therefore, *φE* exposed people become informed people in unit time.

We assume that the potential proportion of active informed people is *p*, and that of inactive informed people is (1−*p*). From the definition of symptom, the two cannot be distinguished in compartment *S* and *E*, and only in the informed compartment can they be distinguished. Therefore, from compartment *E*, *pφE* people flow to compartment *I*_+_ and (1−*p*)*φE* people flow to compartment *I*_−_ in unit time.

We assume the active informed people outflow the system at a constant rate of *d*. Therefore, *dI*_+_ people flow out of the system from compartment *I*_+_ in unit time.

The informed people will recover from the information in various ways and become recovered people. The recovering rate of informed people is denoted by *ω*, measuring the amount of recovery resources. This paper further proposes the normalization of the recovering rate in the two informed compartments. Suppose the primary way to recover the informed people is to be exposed to the recovery resources (e.g., rumor refutation notice) on an information platform and the cost of such resources is fixed. In this way, the people’s recovering rate in two informed compartments is normalized, different from the epidemic models. We denote the proportion of the recovery resources assigned to the active informed people by *q*, so the recovering rate of the active informed people is *qω*, and that assigned to the inactive informed people by (1−*q*), so the recovering rate of the active informed people is (1−*q*)*ω*. Therefore, *qωI*_+_ people flow from compartment *I*_+_ to compartment *R*, and (1−*q*)*ωI*_−_ people flow from compartment *I*_−_ to compartment *R* in unit time.

To ensure the system’s openness, we assume *Λ* people flow into the system per unit time by natural birth. Since the information is not biologically inherited, the *Λ* people flow into compartment *S*. Moreover, we assume the people in the system naturally die at a rate of *μ*. Therefore, *μS*, *μE*, *μI*_+_, *μI*_−_, and *μR* people flow out of the system from compartment *S*, *E*, *I*_+_, *I*_−_, and *R* respectively.

Based on the discussion above, the following nonlinear system is determined.


$$\{\begin{array}{l} \dot{S}=\Lambda -\alpha (I_{+}+I_{-})S-\mu S,\\ \dot{E}=\alpha (I_{+}+I_{-})S-\varphi E-\mu E,\\ {\dot{I}}_{+}=p\varphi E-q\omega I_{+}-(\mu +d)I_{+},\\ {\dot{I}}_{-}=(1-p)\varphi E-(1-q)\omega I_{-}-\mu I_{-},\\ \dot{R}=q\omega I_{+}+(1-q)\omega I_{-}-\mu R. \end{array}$$ (1)

The domain of the parameters in system (1) is stipulated as *Λ*,*μ*,*α*,*φ*,*ω*∈(0,+∞), *d*∈[0,+∞), *p*,*q*∈[0,1]. System (1) degenerates to a classical SEIR epidemic model [15], either when *p* = *q* = 0 or *p* = *q* = 1, *d* = 0.

**Proposition 2.1.** The region

$$\Gamma =\{(S,E,I_{+},I_{-},R)\in R_{\geq 0}^{5}:S+E+I_{+}+I_{-}+R\leq \frac{\Lambda }{\mu }\}$$ (2)

is a compact positive invariant set of system (1) and is attracting within $R_{\geq 0}^{5}$.

**Proof.** First, we prove $R_{\geq 0}^{5}$ is positive invariant; that is, for any positive initial conditions, the variables of system (1) remain positive. (i) $\dot{S}=\Lambda >0$, if *S* = 0. (ii) $\dot{E}=\alpha (I_{+}+I_{-})S\geq 0$, if *E* = 0. (iii) ${\dot{I}}_{+}=p\varphi E\geq 0$, if *I*_+_ = 0. (iv) ${\dot{I}}_{-}=(1-p)\varphi E\geq 0$, if *I*_−_ = 0. (v) $\dot{R}=q\omega I_{+}+(1-q)\omega I_{-}\geq 0$, if *R* = 0. Therefore, we have *E*,*I*_+_,*I*_−_,*R*≥0 and *S*>0, such that $R_{\geq 0}^{5}$ is positive invariant [22].

Secondly, we denote *N* = *S*+*E*+*I*_+_+*I*_−_+*R*. Then,

$$\dot{N}=\dot{S}+\dot{E}+{\dot{I}}_{+}+{\dot{I}}_{-}+\dot{R}=\Lambda -\mu N-dI_{+}\leq \Lambda -\mu N.$$ (3)

Using a standard comparison theorem [39], the following is solved.


$$N\leq ({N|}_{t=0}-\frac{\Lambda }{\mu })e^{-\mu t}+\frac{\Lambda }{\mu }.$$ (4)

Therefore, for *N*|_*t* = 0_≤*Λ*/*μ*, we have *N*≤*Λ*/*μ*, such that *Γ* is positive invariant. In addition, within in $R_{\geq 0}^{5},$ lim_*t*→∞_ *N* = *Λ*/*μ*, such that *Γ* is attracting within $R_{\geq 0}^{5}$.

## 3. Stability analysis

We denote a column vector ***Ψ*** = (*S*,*E*,*I*_+_,*I*_−_,*R*)^*T*^. When system (1) achieves equilibrium, it satisfies $\dot{\Psi }=O$; that is,

$$\{\begin{array}{l} 0=\Lambda -\alpha (I_{+}+I_{-})S-\mu S,\\ 0=\alpha (I_{+}+I_{-})S-\varphi E-\mu E,\\ 0=p\varphi E-q\omega I_{+}-(\mu +d)I_{+},\\ 0=(1-p)\varphi E-(1-q)\omega I_{-}-\mu I_{-},\\ 0=q\omega I_{+}+(1-q)\omega I_{-}-\mu R. \end{array}$$ (5)

Solving Eq (5), we obtain two equilibrium points, denoted by Ψ* and Ψ**. Below, we respectively study the stability of them.

### 3.1. The information-free equilibrium

One of the two solutions of Eq (5) is

$$\Psi^{*}={\left(S^{*},E^{*},{I_{+}}^{*},{I_{-}}^{*},R^{*}\right)}^{T}={\left(\frac{\Lambda }{\mu },0,0,0,0\right)}^{T}.$$ (6)

In Eq (6), E* = *I*_+_* = *I*_−_* = 0; therefore, we can label the equilibrium point Ψ* as the information-free equilibrium point [11].

#### 3.1.1. Local asymptotical stability of the information-free equilibrium

We follow the method proposed by van den Driessche and Watmough [11] in this subsection. The compartment *E*, *I*_+_, and *I*_−_ are defined as the compartments of injurious information. Then, let us find the basic reproduction number $R_{0}$ of injurious information. We separate system (1) into $\dot{\Psi }=F-V$, where

$$F=(\begin{array}{c} F_{S}\\ F_{E}\\ F_{I_{+}}\\ F_{I_{-}}\\ F_{R} \end{array})=(\begin{array}{c} 0\\ \alpha (I_{+}+I_{-})S\\ 0\\ 0\\ 0 \end{array}),$$ (7)

$$V=(\begin{array}{c} V_{S}\\ V_{E}\\ V_{I_{+}}\\ V_{I_{-}}\\ V_{R} \end{array})=(\begin{array}{c} -\Lambda +\alpha (I_{+}+I_{-})S+\mu S\\ \varphi E+\mu E\\ -p\varphi E+q\omega I_{+}+(\mu +d)I_{+}\\ -(1-p)\varphi E+(1-q)\omega I_{-}+\mu I_{-}\\ -q\omega I_{+}-(1-q)\omega I_{-}+\mu R \end{array}).$$ (8)

Taking the compartments of injurious information only, we deduce the following matrix at the information-free equilibrium Ψ*.


$$F=(\begin{array}{ccc} \frac{\partial F_{E}}{\partial E} & \frac{\partial F_{E}}{\partial I_{+}} & \frac{\partial F_{E}}{\partial I_{-}}\\ \frac{\partial F_{I_{+}}}{\partial E} & \frac{\partial F_{I_{+}}}{\partial I_{+}} & \frac{\partial F_{I_{+}}}{\partial I_{-}}\\ \frac{\partial F_{I_{-}}}{\partial E} & \frac{\partial F_{I_{-}}}{\partial I_{+}} & \frac{\partial F_{I_{-}}}{\partial I_{-}} \end{array})(\Psi^{*})=\frac{\alpha \Lambda }{\mu }(\begin{array}{ccc} 0 & 1 & 1\\ 0 & 0 & 0\\ 0 & 0 & 0 \end{array}),$$ (9)

$$V=(\begin{array}{ccc} \frac{\partial V_{E}}{\partial E} & \frac{\partial V_{E}}{\partial I_{+}} & \frac{\partial V_{E}}{\partial I_{-}}\\ \frac{\partial V_{I_{+}}}{\partial E} & \frac{\partial V_{I_{+}}}{\partial I_{+}} & \frac{\partial V_{I_{+}}}{\partial I_{-}}\\ \frac{\partial V_{I_{-}}}{\partial E} & \frac{\partial V_{I_{-}}}{\partial I_{+}} & \frac{\partial V_{I_{-}}}{\partial I_{-}} \end{array})(\Psi^{*})=(\begin{array}{ccc} \varphi +\mu & 0 & 0\\ -p\varphi & q\omega +\mu +d & 0\\ -(1-p)\varphi & 0 & (1-q)\omega +\mu \end{array}).$$ (10)

Then,

$${F∙V}^{-1}=\frac{\alpha \Lambda }{\mu }(\begin{array}{ccc} \frac{\varphi }{\varphi +\mu }\left(\frac{p}{\mu +d+q\omega }+\frac{1-p}{\mu +(1-q)\omega }\right) & \frac{1}{\mu +d+q\omega } & \frac{1}{\mu +(1-q)\omega }\\ 0 & 0 & 0\\ 0 & 0 & 0 \end{array}).$$ (11)

The basic reproduction number $R_{0}$ is the spectral radius of the matrix ***F***∙***V***^−1^. Therefore, we have

$$\begin{array}{c} R_{0}=\frac{\alpha \varphi \Lambda }{\mu (\varphi +\mu )}(\frac{p}{\mu +d+q\omega }+\frac{1-p}{\mu +(1-q)\omega }). \end{array}$$ (12)

where we can see that, $R_{0}$ degenerates to the basic reproduction number of the classical SEIR model [15] (i.e., $R_{0}=\alpha \varphi \Lambda /[\mu (\varphi +\mu )(\mu +\omega )]$) either when *p* = *q* = 0 or *p* = *q* = 1, *d* = 0.

Based on the basic reproduction number $R_{0}$, we have the following theorem.

**Theorem 3.1.1.** The information-free equilibrium Ψ* is locally asymptotically stable, if $R_{0}<1$. The information-free equilibrium Ψ* is not stable, if $R_{0}>1$. The stability of Ψ* requires further study, if $R_{0}=0$.

#### 3.1.2. Global asymptotical stability of the information-free equilibrium

As seen in system (1), the dynamics of *S*, *E*, *I*_+_, and *I*_−_ is independent of *R*; therefore, the stability of variable *S*, *E*, *I*_+_, and *I*_−_ can be determined without the consideration of *R*. We denote

$$\Phi ={\left(S,E,I_{+},I_{-}\right)}^{T},$$ (13)

Then, the stability of ***Ψ*** is equivalent to: (i) The stability of ***Φ***. (ii) The stability of *R*, given ***Φ*** having achieved stability.

Instead of two exposed compartments (i.e., *E*_1_ and *E*_2_) in some previous two-strain epidemic models [22, 23], our model has only one exposed compartmen t *E*. The model is simplified, but the construction of Lyapunov functions becomes more complicated. We must manage to construct coefficients (including Q expressed in Eq (15)) in front of some key variables (e.g., *I*_+_ and *I*−).

**Theorem 3.1.2.** The information-free equilibrium Ψ* is globally asymptotically stable in $R_{\geq 0}^{5}$, if $R_{0}\leq 1$.

**Proof.** Consider the following Lyapunov function in $R_{\geq 0}^{4}$.

$$V_{1}(\Phi )=\frac{{\left(S-S^{*}\right)}^{2}}{2S^{*}}+E+\frac{1}{Q}(\frac{\mu +\varphi }{\varphi })(\frac{I_{+}}{\mu +d+q\omega }+\frac{I_{-}}{\mu +(1-q)\omega }),$$ (14)

where

$$Q=\frac{p}{\mu +d+q\omega }+\frac{1-p}{\mu +(1-q)\omega }.$$ (15)

It is easy to verify: (i) *V*_1_(***Φ***) = 0, if ***Φ*** = Φ*. (ii) *V*_1_(***Φ***)>0, if ***Φ*** ≠ Φ*. Therefore, *V*_1_(***Φ***) is positive definite in the neighborhood of ***Φ*** = Φ*. Secondly, we have

$$\begin{array}{l} {\dot{V}}_{1}(\Phi )=(\frac{S}{S^{*}}-1)\dot{S}+\dot{E}+\frac{1}{Q}(\frac{\mu +\varphi }{\varphi })\frac{{\dot{I}}_{+}}{\mu +d+q\omega }+\frac{1}{Q}(\frac{\mu +\varphi }{\varphi })\frac{{\dot{I}}_{-}}{\mu +(1-q)\omega }\\ \;=\frac{S}{S^{*}}\Lambda -\frac{S}{S^{*}}\alpha (I_{+}+I_{-})S-\frac{S}{S^{*}}\mu S-[\Lambda -\alpha (I_{+}+I_{-})S-\mu S]+\alpha (I_{+}+I_{-})S-\varphi E-\mu E\\ \;+\frac{1}{Q}(\frac{\mu +\varphi }{\varphi })\frac{p\varphi E-q\omega I_{+}-(\mu +d)I_{+}}{\mu +d+q\omega }+\frac{1}{Q}(\frac{\mu +\varphi }{\varphi })\frac{(1-p)\varphi E-(1-q)\omega I_{-}-\mu I_{-}}{\mu +(1-q)\omega }\\ \;=\frac{S}{S^{*}}\Lambda -\frac{\alpha (I_{+}+I_{-})}{S^{*}}S^{2}-\frac{\mu }{S^{*}}S^{2}-\Lambda +\frac{\alpha (I_{+}+I_{-})}{S^{*}}SS^{*}+\frac{\mu }{S^{*}}SS^{*}+\frac{\alpha (I_{+}+I_{-})}{S^{*}}SS^{*}\\ \;-(\varphi +\mu )E+\frac{1}{Q}(\mu +\varphi )∙QE-\frac{1}{Q}(\frac{\mu +\varphi }{\varphi })I_{+}-\frac{1}{Q}(\frac{\mu +\varphi }{\varphi })I_{-} \end{array}$$ (16)

Then, *E* is eliminated. According to Eq (6), we have *Λ* = μS*, such that

$$\begin{array}{l} {\dot{V}}_{1}(\Phi )=\frac{\mu }{S^{*}}SS^{*}-\frac{\alpha (I_{+}+I_{-})}{S^{*}}S^{2}-\frac{\mu }{S^{*}}S^{2}-\frac{\mu }{S^{*}}{S^{*}}^{2}+\frac{\alpha (I_{+}+I_{-})}{S^{*}}SS^{*}+\frac{\mu }{S^{*}}SS^{*}+\frac{\alpha (I_{+}+I_{-})}{S^{*}}SS^{*}\\ \;-\frac{1}{Q}(\frac{\mu +\varphi }{\varphi })(I_{+}+I_{-})\\ \;=-\frac{\mu }{S^{*}}{\left(S-S^{*}\right)}^{2}-\frac{\alpha (I_{+}+I_{-})}{S^{*}}S^{2}+2\frac{\alpha (I_{+}+I_{-})}{S^{*}}SS^{*}+(-\frac{\alpha (I_{+}+I_{-})}{S^{*}}{S^{*}}^{2}+\frac{\alpha (I_{+}+I_{-})}{S^{*}}{S^{*}}^{2})\\ \;-\frac{1}{Q}(\frac{\mu +\varphi }{\varphi })(I_{+}+I_{-})\\ \;=-\frac{\mu }{S^{*}}{\left(S-S^{*}\right)}^{2}-\frac{\alpha (I_{+}+I_{-})}{S^{*}}{\left(S-S^{*}\right)}^{2}+\frac{\alpha \Lambda }{\mu }(I_{+}+I_{-})-\frac{1}{Q}(\frac{\mu +\varphi }{\varphi })(I_{+}+I_{-})\\ \;=-\frac{\mu }{S^{*}}{\left(S-S^{*}\right)}^{2}-\frac{\alpha (I_{+}+I_{-})}{S^{*}}{\left(S-S^{*}\right)}^{2}+\frac{1}{Q}(\frac{\mu +\varphi }{\varphi })(R_{0}-1)(I_{+}+I_{-}). \end{array}$$ (17)

From Eq (17), we have: (i) ${\dot{V}}_{1}(\Phi )=0$, if ***Φ*** = Φ*. (ii) ${\dot{V}}_{1}(\Phi )\leq 0$, if ***Φ*** ≠ Φ* and $R_{0}\leq 1$. Therefore, ${\dot{V}}_{1}(\Phi )$ is negative semidefinite in the neighborhood of ***Φ*** = Φ*, if $R_{0}\leq 1$.

Thus, it is concluded that, (i) *V*_1_(***Φ***) is positive definite in the neighborhood of ***Φ*** = Φ*; (ii) ${\dot{V}}_{1}(\Phi )$ is negative semidefinite in the neighborhood of ***Φ*** = Φ*, if $R_{0}\leq 1$; in addition, it is obvious that, (iii) ${\dot{V}}_{1}(\Phi )≢0$, ∀***Φ*** in $R_{\geq 0}^{4}$; (iv) *V*_1_(***Φ***)→∞, as ‖***Φ***‖→∞. Hence, from Lasalle’s Invariance Principle [12, 13], Φ* is globally asymptotically stable in $R_{\geq 0}^{4}$, if $R_{0}\leq 1$.

After ***Φ*** achieves stability at Φ*, it is easy to verify the global asymptotical stability of *R* at R* by considering the Lyapunov function *V*(*R*) = *R* and $\dot{V}(R)=-\mu R$ in $R_{\geq 0}^{1}$. In summary, the information-free equilibrium Ψ* is globally asymptotically stable in $R_{\geq 0}^{5}$, if $R_{0}\leq 1$.

### 3.2. The information-endemic equilibrium

The second solution of Eq (5) is

$$\Psi^{**}=(\begin{array}{c} S^{**}\\ E^{**}\\ {I_{+}}^{**}\\ {I_{-}}^{**}\\ R^{**} \end{array})=(\begin{array}{c} \frac{\Lambda }{\mu +\alpha ({I_{+}}^{**}+{I_{-}}^{**})}\\ \frac{\Lambda }{\mu +\varphi }(1-\frac{1}{R_{0}})\\ \frac{p\varphi }{\mu +d+q\omega }E^{**}\\ \frac{(1-p)\varphi }{\mu +(1-q)\omega }E^{**}\\ \frac{q\omega {I_{+}}^{**}+(1-q)\omega {I_{-}}^{**}}{\mu } \end{array}).$$ (18)

We label the equilibrium point Ψ** as the information-endemic equilibrium point. From Eq (18), we have E**>0, if and only if $R_{0}>1$. Given E**, we can uniquely determine *I*_+_** and *I*_−_**; then, S** and R** can be uniquely determined. Therefore, the equilibrium-endemic point Ψ** exists, if and only if $R_{0}>1$.

#### 3.2.1. Local asymptotical stability of the information-endemic equilibrium

To investigate the stability of Ψ**, we write the Jacobian matrix of $\dot{\Phi }$ first.


$$J(\dot{\Phi })=(\begin{array}{cccc} -\alpha (I_{+}+I_{-})-\mu & 0 & -\alpha S & -\alpha S\\ \alpha (I_{+}+I_{-}) & -\varphi -\mu & \alpha S & \alpha S\\ 0 & p\varphi & -q\omega -\mu -d & 0\\ 0 & (1-p)\varphi & 0 & -(1-q)\omega -\mu \end{array}),$$ (19)

The 1st and 2nd leading principal minor of $J(\dot{\Phi })$ at Φ** are calculated in Eq (20) and Eq (21).


$$|-\alpha ({I_{+}}^{**}+{I_{-}}^{**})-\mu |<0,$$ (20)

$$\begin{array}{c} \left|\begin{array}{cc} -\alpha ({I_{+}}^{**}+{I_{-}}^{**})-\mu & 0\\ \alpha ({I_{+}}^{**}+{I_{-}}^{**}) & -\varphi -\mu \end{array}\right|\\ =[\alpha ({I_{+}}^{**}+{I_{-}}^{**})+\mu ](\varphi +\mu )>0. \end{array}$$ (21)

From Eq (18), we have substitutions $\left({I_{+}}^{**}+{I_{-}}^{**})=(\mu /\alpha )(R_{0}-1),\;S^{**}=(\Lambda /\mu )(1/R_{0}\right)$; thereafter, the 3rd leading principal minor of $J(\dot{\Phi })$ at Φ** is calculated in Eq (22).


$$\begin{array}{l} \;\left|\begin{array}{ccc} -\alpha ({I_{+}}^{**}+{I_{-}}^{**})-\mu & 0 & -\alpha S^{**}\\ \alpha ({I_{+}}^{**}+{I_{-}}^{**}) & -\varphi -\mu & \alpha S^{**}\\ 0 & p\varphi & -q\omega -\mu -d \end{array}\right|\\ =\mu (\varphi +\mu )(\mu +d+q\omega )(\frac{1}{1+(\frac{1-p}{p})\frac{\mu +d+q\omega }{\mu +(1-q)\omega }}-R_{0})\\ \leq \mu (\varphi +\mu )(\mu +d+q\omega )(1-R_{0})<0. \end{array}$$ (22)

For the 4th leading principal minor of $J(\dot{\Phi })$ at Φ**, we expand it on the fourth row, and it is calculated in Eq (23).


$$\begin{array}{l} \;\left|\begin{array}{cccc} -\alpha ({I_{+}}^{**}+{I_{-}}^{**})-\mu & 0 & -\alpha S^{**} & -\alpha S^{**}\\ \alpha ({I_{+}}^{**}+{I_{-}}^{**}) & -\varphi -\mu & \alpha S^{**} & \alpha S^{**}\\ 0 & p\varphi & -q\omega -\mu -d & 0\\ 0 & (1-p)\varphi & 0 & -(1-q)\omega -\mu \end{array}\right|\\ =(1-p)\varphi |\begin{array}{ccc} -\alpha ({I_{+}}^{**}+{I_{-}}^{**})-\mu & -\alpha S^{**} & -\alpha S^{**}\\ \alpha ({I_{+}}^{**}+{I_{-}}^{**}) & \alpha S^{**} & \alpha S^{**}\\ 0 & -q\omega -\mu -d & 0 \end{array}|\\ \;-[(1-q)\omega +\mu ]|\begin{array}{ccc} -\alpha ({I_{+}}^{**}+{I_{-}}^{**})-\mu & 0 & -\alpha S^{**}\\ \alpha ({I_{+}}^{**}+{I_{-}}^{**}) & -\varphi -\mu & \alpha S^{**}\\ 0 & p\varphi & -q\omega -\mu -d \end{array}|\\ =\mu (\varphi +\mu )(\mu +d+q\omega )\{-\frac{1}{Q}(1-p)+[\mu +(1-q)\omega ](R_{0}-\frac{1}{Q}\frac{p}{\mu +d+q\omega })\}\\ =\mu (\varphi +\mu )(\mu +d+q\omega )[\mu +(1-q)\omega ](R_{0}-1)>0. \end{array}$$ (23)

According to Hurwitz’s theorem, by Eq (20) ~ Eq (23), $J(\dot{\Phi })$ is negative definite in the neighborhood of ***Φ*** = Φ**, if $R_{0}>1$; therefore, Φ** is locally asymptotically stable, if $R_{0}>1$. After ***Φ*** achieves stability at Φ**, we write the Jacobian matrix $J(\dot{R})=-\mu R$, and it is easy to verify $J(\dot{R})$ is negative definite in the neighborhood of *R* = R**; therefore, R** is locally asymptotically stable. In summary, we have the following theorem.

**Theorem 3.2.1.** The information-endemic equilibrium Ψ** is locally asymptotically stable, if $R_{0}>1$. The information-endemic equilibrium Ψ** is not stable, if $R_{0}\leq 1$.

#### 3.2.2. Global asymptotical stability of the information-endemic equilibrium

Having the stability of the equilibrium, we now study the global stability.

**Theorem 3.2.2.** The stable information-endemic equilibrium Ψ** is globally asymptotically stable in $R_{\geq 0}^{5}$.

**Proof.** Consider the following Lyapunov function in $R_{\geq 0}^{4}$.


$$\begin{array}{l} V_{2}(\Phi )=S^{**}[\frac{S}{S^{**}}-(1+\ln\frac{S}{S^{**}})]+E^{**}[\frac{E}{E^{**}}-(1+\ln\frac{E}{E^{**}})]\\ \;+\frac{1}{Q}(\frac{\mu +\varphi }{\varphi })\{\left(\frac{{I_{+}}^{**}}{\mu +d+q\omega })[\frac{I_{+}}{{I_{+}}^{**}}-(1+\ln\frac{I_{+}}{{I_{+}}^{**}})]+(\frac{{I_{-}}^{**}}{\mu +(1-q)\omega })[\frac{I_{-}}{{I_{-}}^{**}}-(1+\ln\frac{I_{-}}{{I_{-}}^{**}})\right]\} \end{array}$$ (24)

It is easy to verify: (i) *V*_2_(***Φ***) = 0, if ***Φ*** = Φ**. (ii) *V*_2_(***Φ***)>0, if ***Φ*** ≠ Φ**. Therefore, *V*_2_(***Φ***) is positive definite in the neighborhood of ***Φ*** = Φ**. Secondly, we have

$$\begin{array}{l} {\dot{V}}_{2}(\Phi )=(1-\frac{S^{**}}{S})\dot{S}+(1-\frac{E^{**}}{E})\dot{E}+\frac{1}{Q}(\frac{\mu +\varphi }{\varphi })[\frac{(1-\frac{{I_{+}}^{**}}{I_{+}}){\dot{I}}_{+}}{\mu +d+q\omega }+\frac{(1-\frac{{I_{-}}^{**}}{I_{-}}){\dot{I}}_{-}}{\mu +(1-q)\omega }]\\ \;=\Lambda -\alpha (I_{+}+I_{-})S-\mu S-\frac{S^{**}}{S}\Lambda +\alpha (I_{+}+I_{-})S^{**}+\mu S^{**}+\alpha (I_{+}+I_{-})S-\varphi E-\mu E\\ \;-\alpha E^{**}(I_{+}+I_{-})\frac{S}{E}+(\varphi +\mu )E^{**}+\frac{\mu +\varphi }{Q}(\frac{p}{\mu +d+q\omega })E-\frac{1}{Q}(\frac{\mu +\varphi }{\varphi })I_{+}\\ \;-\frac{\mu +\varphi }{Q}(\frac{p}{\mu +d+q\omega }){I_{+}}^{**}\frac{E}{I_{+}}+\frac{1}{Q}(\frac{\mu +\varphi }{\varphi }){I_{+}}^{**}+\frac{\mu +\varphi }{Q}(\frac{1-p}{\mu +(1-q)\omega })E-\frac{1}{Q}(\frac{\mu +\varphi }{\varphi })I_{-}\\ \;-\frac{\mu +\varphi }{Q}(\frac{1-p}{\mu +(1-q)\omega }){I_{-}}^{**}\frac{E}{I_{-}}+\frac{1}{Q}(\frac{\mu +\varphi }{\varphi }){I_{-}}^{**}\\ \;=\Lambda -\mu S-\frac{S^{**}}{S}\Lambda +\alpha (I_{+}+I_{-})S^{**}+\mu S^{**}-\alpha E^{**}(I_{+}+I_{-})\frac{S}{E}+(\varphi +\mu )E^{**}\\ \;+\frac{1}{Q}(\frac{\mu +\varphi }{\varphi })[\left({I_{+}}^{**}+{I_{-}}^{**})-(I_{+}+I_{-}\right)]-\frac{\mu +\varphi }{Q}E[(\frac{p}{\mu +d+q\omega })\frac{{I_{+}}^{**}}{I_{+}}+(\frac{1-p}{\mu +(1-q)\omega })\frac{{I_{-}}^{**}}{I_{-}}] \end{array}$$ (25)

From Eq (18), we have substitutions *Λ* = [*μ*+*α*(*I*_+_**+*I*_−_**)]S**, $\alpha S^{**}=(1/Q)(\mu +\varphi )/\varphi ,\;E^{**}=[\alpha /(\mu +\varphi )]({I_{+}}^{**}+{I_{-}}^{**})S^{**}=[\Lambda /(\mu +\varphi )](1-1/R_{0}),\;{I_{+}}^{**}+{I_{-}}^{**}=(\mu /\alpha )(R_{0}-1)$; thereafter,

$$\begin{array}{l} {\dot{V}}_{2}(\Phi )=\mu S^{**}+\alpha ({I_{+}}^{**}+{I_{-}}^{**})S^{**}-\mu S^{**}\frac{S}{S^{**}}-\mu S^{**}\frac{S^{**}}{S}-\alpha ({I_{+}}^{**}+{I_{-}}^{**})S^{**}\frac{S^{**}}{S}+\alpha (I_{+}+I_{-})S^{**}\\ \;+\mu S^{**}-\alpha ({I_{+}}^{**}+{I_{-}}^{**})S^{**}\frac{SE^{**}(I_{+}+I_{-})}{S^{**}E({I_{+}}^{**}+{I_{-}}^{**})}+\alpha ({I_{+}}^{**}+{I_{-}}^{**})S^{**}+\alpha ({I_{+}}^{**}+{I_{-}}^{**})S^{**}\\ \;-\alpha (I_{+}+I_{-})S^{**}-\frac{\mu +\varphi }{Q}E[(\frac{p}{\mu +d+q\omega })\frac{{I_{+}}^{**}}{I_{+}}+(\frac{1-p}{\mu +(1-q)\omega })\frac{{I_{-}}^{**}}{I_{-}}]\\ \;=\mu S^{**}(2-\frac{S}{S^{**}}-\frac{S^{**}}{S})+\alpha ({I_{+}}^{**}+{I_{-}}^{**})S^{**}(3-\frac{S^{**}}{S}-\frac{SE^{**}(I_{+}+I_{-})}{S^{**}E({I_{+}}^{**}+{I_{-}}^{**})})\\ \;-\alpha ({I_{+}}^{**}+{I_{-}}^{**})S^{**}\frac{E}{QE^{**}}[(\frac{p}{\mu +d+q\omega })\frac{{I_{+}}^{**}}{I_{+}}+(\frac{1-p}{\mu +(1-q)\omega })\frac{{I_{-}}^{**}}{I_{-}}]. \end{array}$$ (26)

According to the Jensen Inequality,

$$(\frac{p}{\mu +d+q\omega })\frac{{I_{+}}^{**}}{I_{+}}+(\frac{1-p}{\mu +(1-q)\omega })\frac{{I_{-}}^{**}}{I_{-}}\geq (\frac{p}{\mu +d+q\omega }+\frac{1-p}{\mu +(1-q)\omega })\frac{{I_{+}}^{**}+{I_{-}}^{**}}{I_{+}+I_{-}}.$$ (27)

Substituting Eq (27) into Eq (26), and considering the Inequality of arithmetic and geometric means, we have the following result.


$$\begin{array}{l} {\dot{V}}_{2}(\Phi )\leq \mu S^{**}(2-\frac{S}{S^{**}}-\frac{S^{**}}{S})+\alpha ({I_{+}}^{**}+{I_{-}}^{**})S^{**}(3-\frac{S^{**}}{S}-\frac{SE^{**}(I_{+}+I_{-})}{S^{**}E({I_{+}}^{**}+{I_{-}}^{**})}-\frac{E({I_{+}}^{**}+{I_{-}}^{**})}{E^{**}(I_{+}+I_{-})})\\ \;\leq 0. \end{array}$$ (28)

From Eq (28), we have: (i) ${\dot{V}}_{2}(\Phi )=0$, if ***Φ*** = Φ**. (ii) ${\dot{V}}_{2}(\Phi )\leq 0$, if ***Φ*** ≠ Φ**. Therefore, ${\dot{V}}_{2}(\Phi )$ is negative semidefinite in the neighborhood of ***Φ*** = Φ**. In addition, ${\dot{V}}_{2}(\Phi )≢0,$ ∀***Φ*** in $R_{\geq 0}^{4}$; *V*_2_(***Φ***)→∞, as ‖***Φ***‖→∞. Hence, by Lasalle’s Invariance Principle [12, 13], Φ** is globally asymptotically stable in $R_{\geq 0}^{4}$.

After ***Φ*** achieves stability at Φ**, it is easy to verify the global asymptotical stability of *R* at R** in $R_{\geq 0}^{1}$. Therefore, the information-endemic equilibrium Ψ** is globally asymptotically stable in $R_{\geq 0}^{5}$.

## 4. Results and discussion

### 4.1. Numerical results

In this subsection, we further illustrate the results of theoretical analysis by numerical results. The numerical results are acquired by the forward-Euler difference method as several similar works did [7, 41]. We fix the step of time evolution as Δ*t* = 10^−2^, and when *t* = Δ*t*, we set the initial conditions *S* = 30, *E* = 0, *I*_+_ = 5, *I*_−_ = 5, *R* = 0. The system evolves from *t* = 10^−2^ to *t* = 10^−4^. We complete the calculation and produce all figures by Matlab 2016a in Windows 10.

**Fig 2:**
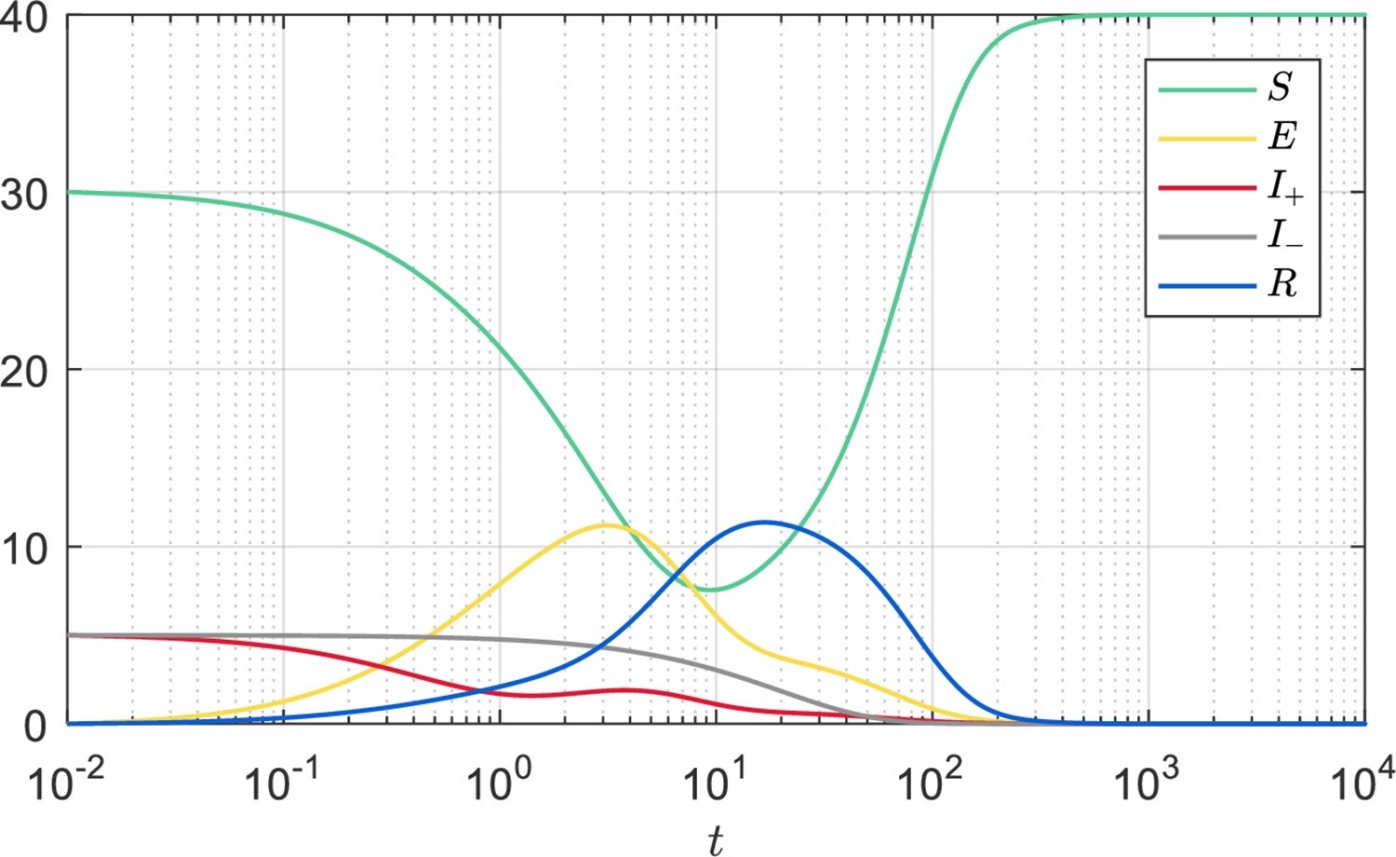
Time evolution of the population. *Λ* = 2, *μ* = 0.05, *d* = 0.9, *α* = 0.05, *φ* = 0.3, *ω* = 0.8, *p* = 1, *q* = 1, such that $R_{0}\approx 0.9796<1.$ *S* = 30, *E* = 0, *I*_+_ = 5, *I*_−_ = 5, *R* = 0, when *t* = 10^−2^.

First, we set the system parameters *Λ* = 2, *μ* = 0.05, *d* = 0.9, *α* = 0.05, *φ* = 0.3, *ω* = 0.8, *p* = 1, *q* = 1, and the time evolution of system (1) is shown in Fig 2. From Eq (12), it is calculated that $R_{0}\approx 0.9796<1$. According to Theorem 3.1.1, the information-free equilibrium Ψ* is locally asymptotically stable, and according to Theorem 3.2.1, the information-endemic equilibrium Ψ** is not stable. Through Eq (6), we have Ψ* = (S*,E*,*I*_+_*,*I*_−_*,R*)^*T*^ = (*Λ*/*μ*, 0,0,0,0)^*T*^ = (40,0,0,0,0)^*T*^, which is consistent with the numerical result of time evolution in Fig 2. In addition, when *t* = 10^−4^, we have *S*+*E*+*I*_+_+*I*_−_+*R* = 40≤*Λ*/*μ*, which further validates Proposition 2.1.

**Fig 3:**
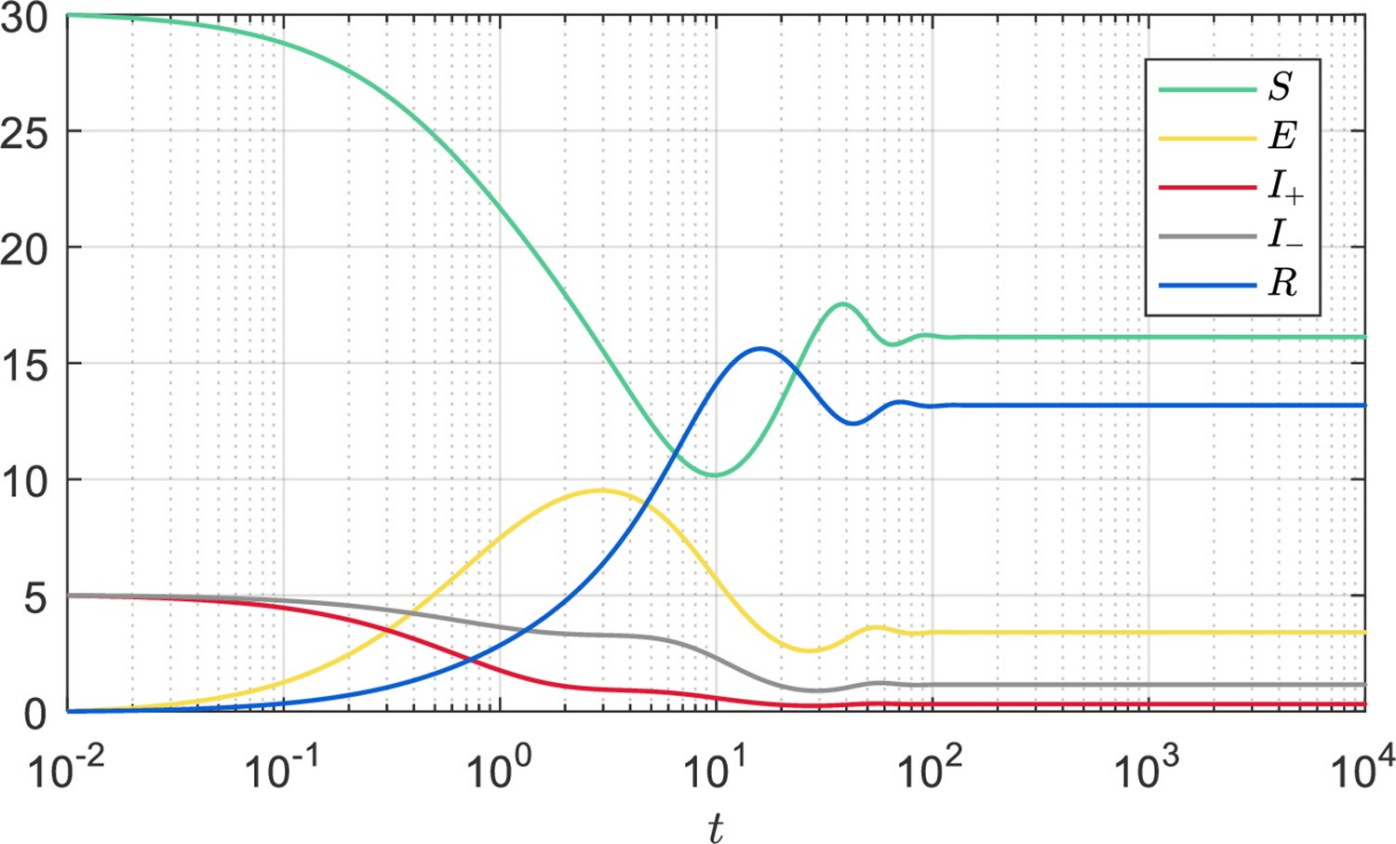
Time evolution of the population. *Λ* = 2, *μ* = 0.05, *d* = 0.9, *α* = 0.05, *φ* = 0.3, *ω* = 0.8, *p* = 0.4, *q* = 0.4, such that $R_{0}\approx 2.4806>1.$ *S* = 30, *E* = 0, *I*_+_ = 5, *I*_−_ = 5, *R* = 0, when *t* = 10^−2^.

Secondly, we set the system parameters *Λ* = 2, *μ* = 0.05, *d* = 0.9, *α* = 0.05, *φ* = 0.3, *ω* = 0.8, *p* = 0.4, *q* = 0.4, and the time evolution of system (1) is shown in Fig 3. From Eq (12), it is calculated that $R_{0}\approx 2.4806>1$. According to Theorem 3.1.1, the information-free equilibrium Ψ* is not stable, and according to Theorem 3.2.1, the information-endemic equilibrium Ψ** is locally asymptotically stable. Through Eq (18), we have S** ≈ 16.1249, E** ≈ 3.4107, *I*_+_* ≈ 0.3223, *I*_−_* ≈ 1.1584, R* ≈ 13.1828, which is consistent with the numerical result of time evolution in Fig 3. Moreover, when *t* = 10^4^, we have *S*+*E*+*I*_+_+*I*_−_+*R* ≈ 34.1991≤*Λ*/*μ*, which is also consistent with Proposition 2.1.

### 4.2. Discussion on removing the injurious information

We now study the strategies of removing the injurious information by analyzing its basic reproduction number $R_{0}$ [33, 40]. As introduced in Section 2, parameter *q* represents the proportion of recovery resources assigned to the active informed people with recovery resources fixed. In contrast, (1−*q*) represents that assigned to the inactive informed ones. The goal is to minimize $R_{0}$ by adjusting *q*.

Therefore, we have the first derivative of the basic reproduction number $R_{0}$ to the parameter *q*,

$$\frac{\partial R_{0}}{\partial q}=\frac{\alpha \varphi \Lambda \omega }{\mu (\varphi +\mu )}\{-\frac{p}{{\left(\mu +d+q\omega \right)}^{2}}+\frac{1-p}{{\left[\mu +(1-q)\omega \right]}^{2}}\},$$ (29)

and the second derivative of the basic reproduction number $R_{0}$ to the parameter *q*

$$\frac{\partial^{2}R_{0}}{\partial q^{2}}=\frac{2\alpha \varphi \Lambda \omega^{2}}{\mu (\varphi +\mu )}\{\frac{p}{{\left(\mu +d+q\omega \right)}^{3}}+\frac{1-p}{{\left[\mu +(1-q)\omega \right]}^{3}}\}>0.$$ (30)

From Eq (30), $\partial R_{0}/\partial q$ increases monotonously with the increase of *q*; after that, we discuss three cases considering 0≤*q*≤1 below.

First, we have $\partial R_{0}/\partial q\geq 0$ when

$${\frac{\partial R_{0}}{\partial q}|}_{q=0}=\frac{\alpha \varphi \Lambda \omega }{\mu (\varphi +\mu )}[-\frac{p}{{\left(\mu +d\right)}^{2}}+\frac{1-p}{{\left(\mu +\omega \right)}^{2}}]\geq 0.$$ (31)

Solving Eq (31), we have

$$p\leq \frac{1}{1+{\left(\frac{\mu +\omega }{\mu +d}\right)}^{2}}≔p^{\left(0\right)}.$$ (32)

When Eq (32) is satisfied, $R_{0}$ increases monotonously with the increase of *q*; therefore, $R_{0}$ is the minimum, when *q* = *q*_0_ = 0.

Secondly, we have $\partial R_{0}/\partial q\leq 0$ when

$${\frac{\partial R_{0}}{\partial q}|}_{q=1}=\frac{\alpha \varphi \Lambda \omega }{\mu (\varphi +\mu )}[-\frac{p}{{\left(\mu +d+q\omega \right)}^{2}}+\frac{1-p}{\mu^{2}}]\leq 0.$$ (33)

Solving Eq (33), we have

$$p\geq \frac{1}{1+{\left(\frac{\mu }{\mu +d+\omega }\right)}^{2}}≔p^{\left(1\right)}.$$ (34)

When Eq (34) is satisfied, $R_{0}$ decreases monotonously with the increase of *q*; therefore, $R_{0}$ is the minimum, when *q* = *q*_0_ = 1.

The third case is

$$\{\begin{array}{c} {\frac{\partial R_{0}}{\partial q}|}_{q=0}<0,\\ {\frac{\partial R_{0}}{\partial q}|}_{q=1}>0. \end{array}$$ (35)

Solving Eq (33), we have

$$p^{\left(0\right)}<p<p^{\left(1\right)}.$$ (36)

In this case, $\partial R_{0}/\partial q$ has a zero point *q*_0_ in the interval [0,1]. Let $\partial R_{0}/\partial q=0$, then we solve *q*_0_ as

$$q_{0}=\frac{1}{\omega }∙\frac{(\mu +\omega )\sqrt{p}-(\mu +d)\sqrt{1-p}}{\sqrt{p}+\sqrt{1-p}}.$$ (37)

When *q*<*q*_0_, $\partial R_{0}/\partial q<0,\;R_{0}$ decreases monotonously with the increase of *q*, and when *q*>*q*_0_, $\partial R_{0}/\partial q>0,\;R_{0}$ increases monotonously with the increase of *q*. When *q* = *q*_0_, $\partial R_{0}/\partial q=0,\;R_{0}$ is the minimum.

In summary, we have the following expression of the optimal proportion of recovery resources *q*_0_ assigned to the active informed people that minimizes the basic reproduction number of the injurious information $R_{0}$.


$$q_{0}=\{\begin{array}{l} 0,\;p\leq p^{\left(0\right)},\\ \frac{1}{\omega }∙\frac{(\mu +\omega )\sqrt{p}-(\mu +d)\sqrt{1-p}}{\sqrt{p}+\sqrt{1-p}},\;p^{\left(0\right)}<p<p^{\left(1\right)},\\ 1,\;p\geq p^{\left(1\right)}. \end{array}$$ (38)

According to Eq (38), the larger the proportion of potential active people, the more recovery resources should be allocated. Instead of the idealized analytical solution, as qualitative analysis, we further ask: should it pay more attention to both groups of people, or only focus on one compartment? From Eqs (32) and (34), it is easy to know *p*^(0)^>0 and *p*^(1)^<1. Considering 0≤*p*≤1, it shows that, with regard to the different proportion of active informed people, there is not only the situation that both groups of people should be considered but also the situation that only one compartment need emphasized. It is found that, when active informed people exist, it should not always consider their recovery resources allocation (*p*^(0)^>0), but only when their proportion reaches the critical point *p*^(0)^. As the same, when inactive informed people exist, it should not always consider their recovery resources allocation (*p*^(1)^<1), but only when their proportion reaches the critical point (1−*p*^(1)^). Such conclusions are the general outcomes of the nonlinear dynamics.

However, the practical significance of parameter size still deserves further consideration. For instance, the natural mortality of population *μ*, whose value is usually minimal, is a secondary parameter in the information transmission scenarios we study; therefore, let us say, *μ* = 0.01. In the scenarios of information propagation, the recovering rate *ω*, and the rate of the active informed people leaving the system *d* are the main parameters we are concerned about. Therefore, in general, there should be *ω*≫*μ*, *d*≫*μ*.

**Fig 4:**
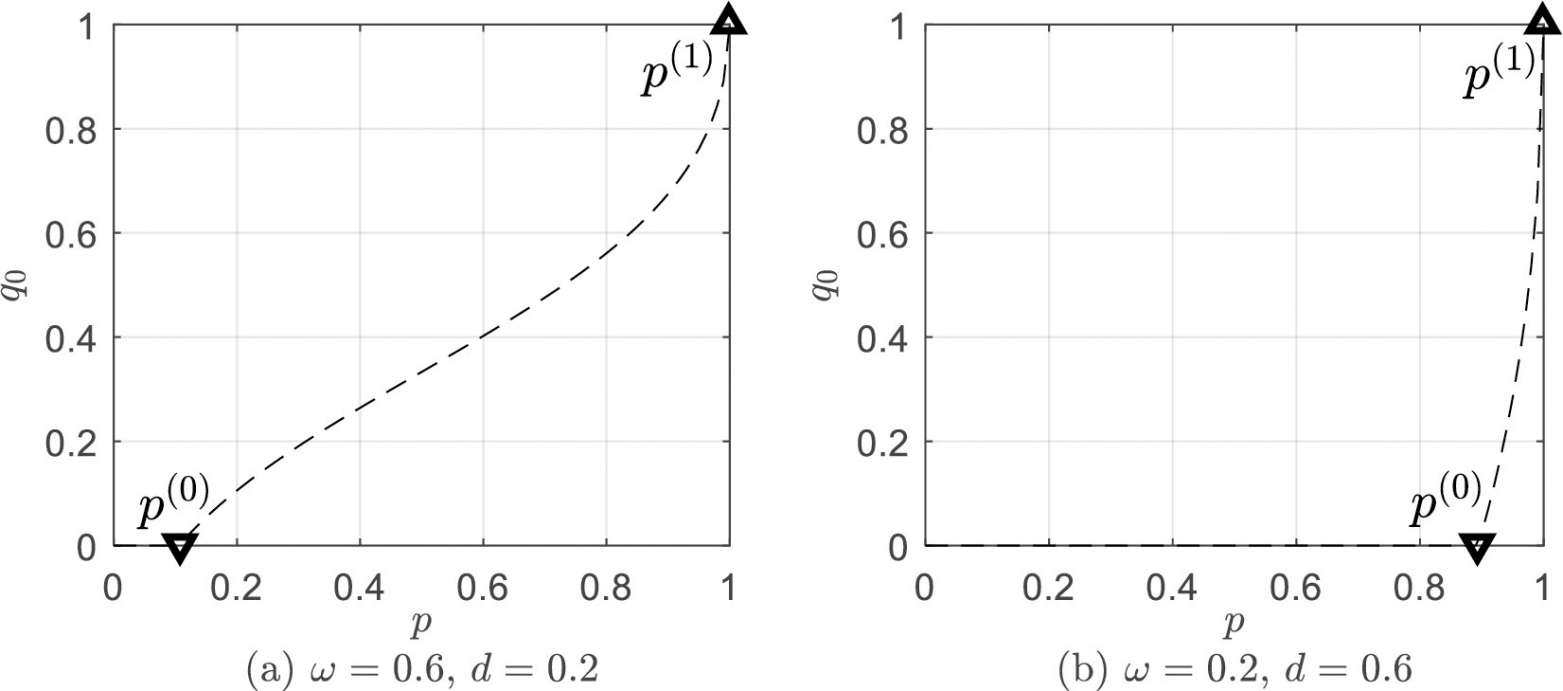
The optimal proportion of recovery resources *q*_0_ assigned to the active informed people as a function of *p*, when *μ* = 0.01. (a) *ω* = 0.6, *d* = 0.2. (b) *ω* = 0.2, *d* = 0.6.

Fig 4 demonstrates the optimal proportion of recovery resources *q*_0_ assigned to the active informed people as a function of *p*, when *μ* = 0.01. Considering *ω*≫*μ*, *d*≫*μ* in Eq (34), we have *p*^(1)^→1^−^, which means that, the critical point of the proportion of inactive people is very low, almost once there are some inactive informed people, they should be allocated recovery resources. As seen in Fig 4, *p*^(1)^ is very close to 1.

Fig 4A shows the situation when *ω* = 0.6, *d* = 0.2, and Fig 4B illustrates that when *ω* = 0.2, *d* = 0.6. According to Eq (32), we have *p*^(0)^→1^+^, when *w*≫*d*; and, we have *p*^(0)^→1^−^, when *w*≪*d*. In other words, when the recovering rate of the population is much higher than the rate of active informed people leaving the system, the critical point of the proportion of active people is low. Once there are some active informed people, they should be allocated recovery resources (Fig 4A). When the rate of active informed people leaving the system is much higher than the recovering rate of the population, the critical point of the proportion of active people is greater, and only when there are a certain number of active informed people should they be allocated recovery resources (Fig 4B).

**Fig 5:**
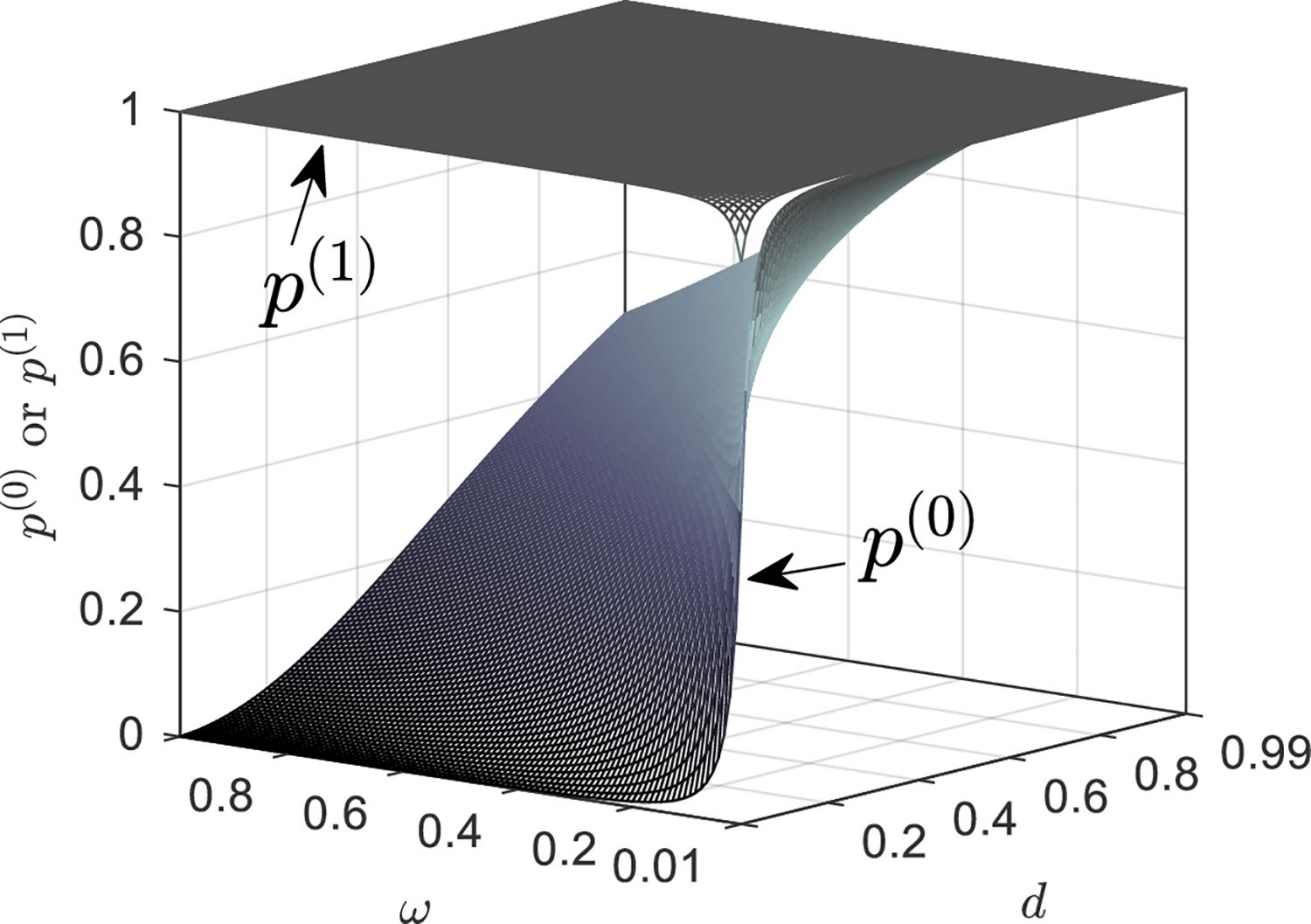
The critical points *p*^(0)^ and *p*^(1)^ as functions of *ω* and *d*, when *μ* = 0.01.

Furthermore, Fig 5 shows *p*^(0)^ and *p*^(1)^ as functions of *ω* and *d*, when *μ* = 0.01. First, the curved surface of *p*^(1)^ is consistent with the analysis of *p*^(1)^→1^−^ when *ω*≫*μ*, *d*≫*μ*. Secondly, *p*^(0)^ varies obviously with the relative size of *ω* and *d*. It can be concluded that an increase in *ω* decreases *p*^(0)^; in other words, when the recovering rate of the informed people rises, more often, we need to focus on both groups of informed people. However, an increase in *d* increases *p*^(0)^; that is, when the rate of the active informed people leaving the system rises, the interval focusing on both the groups becomes smaller, while ignoring active informed people is larger. This is because the outflow system of active informed people is also a way to reduce the informed people, which has played the same role as recovery.

## 5. Conclusion

This paper studied the propagation of injurious information in a nonlinear compartment model and discussed removing the injurious information. The injuriousness or symptom of the injurious information is defined as the people who practice the information being harmed and leaving the system. We assumed a proportion of informed people practice the information, while other informed people do not practice. Unlike previous studies on rumor or extremism, the difference between the two groups of informed people lies in whether they practice the information, not whether they accept it. The information resources are different from the medical resources; therefore, given the recovery resources, the two groups’ recovering rate is normalized. By the basic reproduction number [11], the Jacobian matrix, and Lyapunov’s Second Method [12, 13], we gave the proposed nonlinear system complete stability analysis.

The basic reproduction number of the injurious information revealed the qualitative strategies of removing the injurious information. Different from our intuition, when active informed people exist (0<*p*≤*p*^(0)^), it is not always necessary to consider their recovery resource allocation (*q*_0_ = 0). Only when its proportion achieves a critical point *p*^(0)^, should the recovery resources be allocated. The same conclusion applies to inactive informed people in the available parameter space. In common sense, it is significant to prevent the injurious information or the epidemic at the outset, or “guard against the minute.” However, as the analysis reveals, unless a group of informed people form a certain proportion, we can take a laissez faire attitude towards them. With the further consideration of the parameters’ practical significance, the critical point of the proportion of inactive people tends to 0; that is, almost once there are some inactive informed people, they should be allocated recovery resources. Moreover, the critical point of the proportion of active people is related to the informed people’s recovering rate and the rate of active informed people leaving the system. When the informed people’s recovering rate rises, we need to focus on both groups of informed people in a greater parameter range. When the rate of active informed people leaving the system rises, the parameter interval ignoring active informed people becomes larger. Although the simple idealized model cannot predict accurate data for a specific problem, it draws heuristic qualitative conclusions in the scenarios of removing injurious information.

Future work could further investigate the transition between active and inactive informed people, resulting from the change of individual attitude in behavior on practicing the information.
